# The effects of different levels of sports activity on health-related quality of life and lifestyle habits in high school Italian students

**DOI:** 10.1007/s00431-024-05661-w

**Published:** 2024-07-02

**Authors:** Lazzeri Maria Francesca Lodovica, Mastorci Francesca, Piaggi Paolo, Trivellini Gabriele, Casu Anselmo, Devine Caleb, Doveri Cristina, Marinaro Irene, Pingitore Alessandro

**Affiliations:** 1 Clinical Physiology Institute, CNR, Via Moruzzi, 1, 56124 Pisa, Italy; 2 Department of Information Engineering, University of Pisa, Pisa, Italy

**Keywords:** Individual sports, Team sports, Adolescents, HRQoL, Well-being, High school

## Abstract

Physical activity (PA) is an important predictor of physical and mental health preventing chronic degenerative diseases. The purpose of this study was to investigate in a group of Italian high school students whether health-related quality of life (HRQoL) and lifestyle habits (diet) are associated with the level of physical activity performed (low, moderate, high). Data were collected from 2819 adolescents (*n* = 951 males). HRQoL was analyzed using the Italian version of the KIDSCREEN-52. Physical activity level was analyzed using the PAQ-A, while eating habits with KIDMED. Practicing physical activity in general improves HRQoL. Specifically, adolescents practicing moderate or high PA, in single dimensions of HRQoL, showed better mood (*p* < 0.001), self-perception (*p* < 0.001), family relationships (*p* < 0.001), reported a higher perception of socioeconomic status (*p* < 0.05), relationship with peers (*p* < 0.001), and social acceptance (*p* < 0.001). High PA subjects reported increased physical (*p* < 0.001) and mental health (*p* < 0.001), increased autonomy (*p* < 0.001), and school learning (*p* < 0.001). For lifestyle habits, practicing moderate PA showed higher adherence tox the Mediterranean diet (*p* < 0.001).

*Conclusion*: Our results highlighted a positive association between the frequency of PA levels, some dimensions of HRQoL, and risk behaviors. These findings demonstrated the protective role of sports not only as a preventive strategy for the onset of chronic degenerative diseases, but also as an educator of healthy lifestyle habits, thus suggesting the importance and need to implement strategies to promote sports practice.

## Introduction

Physical activity (PA) is an important predictor of health, intended as physical, particularly cardiovascular and skeletal, and mental, contributing to the prevention and the cure of chronic degenerative diseases [1–5]. The protective PA effects become even more useful during certain sensitivity windows, such as adolescence, when regular PA can help improve cardiorespiratory fitness, build strong bones and muscles, control weight, mitigate symptoms of anxiety and depression, and reduce the risk of developing unhealthy conditions (https://www.cdc.gov/healthyschools/physicalactivity/facts.htm#:~:text=Regular%20physical%20activity%20can%20help,developing%20health%20conditions%20such%20as%3A&text=Heart%20disease.,Type%202%20diabetes). Accordingly, the results of a recent systematic review showed that childhood and adolescence, being physically active, had a lower risk of multimorbidity in adulthood, although the certainty of evidence was low [6]. One of the reasons for this is that individuals physically active since youth have a favorable cardiometabolic risk profile in adulthood, and thus lower cardiovascular risk [6]. Moreover, several studies pointed out the relationship between PA intensity and effects on different determinants of health [7–9]. Interestingly, in a systematic review, total PA was favorably associated with physical, psychological/social, and cognitive health indicators. However, the association was more consistent and robust in the young subjects performing moderate-to-vigorous PA intensity in comparison to those performing low PA. This was positively associated with cardiometabolic biomarkers [6]. Currently, the WHO recommends that youth spend a 60 min/day moderate to vigorous intensity PA [10]. Moreover, Merglen A et al. showed a U-shaped relationship between weekly sports practice duration and well-being among adolescents [11]. Wilson OWA et al. showed that the breadth of sports participation, both in terms of the number of sports and sports settings, was associated with higher well-being [12]. In this context, it is important to highlight the possible link between sport activities and quality of life, in particular considering health related quality of life (HRQoL), i.e., quality of life in the setting of one’s health and/or illness. More in detail, the specific dimensions of HRQL considered (e.g., psychological well-being, self-esteem, body image, cognitive functioning, mobility, energy/vitality, social relations, and family/home function) are related to aspects of physical, mental, and social health, and thus well-being. In the study of Bermejo-Cantarero A, PA was effective for improving overall HRQoL and its most significant domains, although there were no differences in the effectiveness of the interventions by category of PA intensity [14]. However, in a previous systematic review, the authors showed that higher levels of PA were associated with better HRQoL [15]. Interestingly, the studies included in that systematic review used different methods to measure PA, such as pedometer, accelerometer or self-reported questionnaires, and different methods to distinguish among low, middle, and high PA. Moreover, there are different determinants of health, including body composition, cardiometabolic biomarkers, physical fitness, social behavior, cognition/academic achievement, and quality of life [15]. Therefore, all these differences may generate discrepancies in results. The hypothesis of this study is that the higher the intensity of PA the higher the benefits in the determinants of HRQoL [15]. To this aim, we used the Physical Activity Questionnaire for Adolescents (PAQ-A) providing us to stratify the response in terms of PA. Therefore, the purpose of this study was to investigate the relationships between different levels of PA (low, medium, high) and HRQoL considering perception of physical and psychological well-being, social relationships, emotional state, and lifestyle habits (diet) in a group of high school Italian students.

## Materials and methods

### Participants and procedures

Data in the AVATAR study were collected between 2022 and 2023 by means of web platform developed by the Institute of Clinical Physiology of the CNR, on a sample of 1908 students [16]. Six high schools, enrolled in the AVATAR project “*A new purpose for promotion and eVAluation of healTh and well-being Among healthy teenageRs*” on a voluntary bases and here presented, are located in North Italy, mainly in Tuscany (*n* = 2) and in Friuli Venezia Giulia (*n* = 4). Adolescent students were enrolled according to the following inclusion criteria: age 10–18 years, absence of neuropsychiatric or other diseases, informed consent signed, and filling of the entire questionnaires proposed. Of the initial population of 2901, 82 students were excluded for the following reasons: diagnosed neuropsychiatric or other diseases (*n* = 9), absence of sign informed consent (*n* = 48), questionnaires not filled (*n* = 15), and internet connection problems (*n* = 10). Therefore, the final population consisted of 2819 adolescents (mean age 14.9 ± 3.7 years) composed by 951 boys and 1868 girls. Participants were instructed to complete the questionnaires and all tests were performed during school hours. In every school class, all the adolescents filled out the questionnaire, and whether they were not eligible due to exclusion criteria reasons were excluded from the study retrospectively. Participants were previously instructed on how to complete the questionnaires and conduct the tests. One or two project members visited each school to provide the adolescents with verbal and written information about the data collection. All questionnaires were conducted during participants’ computer lessons during school time. No incentive was provided to adolescents or parents. A research assistant was available to provide information and technical support to complete questionnaires.

### Ethics

All procedures performed in the study were by the ethical standards of the institutional and/or national research committee and with the 1964 Helsinki Declaration and its later amendments or comparable ethical standards, in compliance with the Ethics Rules for Processing for Statistical or Scientific Research Purposes of anonymized sensitive data (Legislative Decree No 101 of 10 August 2018—19 December 2018, Garante Order No 515/2018). The AVATAR project has been accepted by the Regional Pediatric Ethics Committee (Azienda Ospedaliero Universitaria Meyer) (code 166/2018).

### Measures

All questionnaires were self-administered by means of AVATAR web platform. The Physical Activity Questionnaire for Adolescents (PAQ-A) was used to assess PA levels.

The questionnaire provides a general measure of PA for 14- to 19-year olds. The PAQ-A is a self-administrated questionnaire consisting of nine items, e.g., “In the last 7 days, during your physical education classes, how often were you very active (playing hard, running, jumping, throwing)?,” “In the last 7 days, on how many evenings did you do sports, dance, or play games in which you were very active?,” “On the last weekend, how many times did you do sports, dance, or play games in which you were very active?” PAQ-A questionnaire provides a summary PA score derived from eight items, each scored on a 5-point scale (from 1 to 5 point). This PA composite score is calculated taking the mean of these 8 items, which results in the final PAQ-A activity summary score. The average of the items is used to create the final PAQ summary score, a higher score indicating more active adolescents [17, 18].

The scoring directions for this test report values such as low physical activity for scores equal to 1 and high physical activity for scores equal to 5. For this reason, and because almost none of our subjects reported values of 5, we calculated the scores differently, as the aim of the work was to assess the intensity of the sports activity and not the total score. Therefore, the following cut-offs of the PAQ-A were used to stratify the population into three levels: value < 2 identifies low PA (LPA) group; values between 2 and 4 identify moderate PA (MPA) group, and values ≥ 4 high PA (HPA) group.

The PAQ-A has been tested for multiple psychometric properties, using adolescents. In separate studies, item and scale properties, test–retest reliability, internal consistency, sensitivity to gender- and age differences, convergent validity, and construct validity have been examined and reported as acceptable to good [13]. With respect to convergent validity, the instrument correlated with physical activity as measured by a Caltrac activity monitor (*r* = 0.33) [17].

To assess HRQoL, the Italian version of KIDSCREEN-52 was used [19, 20]. The KIDSCREEN is a self-report questionnaire to monitor and measure the personal experiences of children and adolescents regarding their perceptions of health status and well-being. The questionnaire, which describes physical, psychological, mental, social, and functional aspects of well-being, consists of 52 items grouped in 10 dimensions (physical well-being, psychological well-being, moods and emotions, self-perception, autonomy, parent relations, and home life, social support and peers, school environment, social acceptance (bullying), financial resources) [19, 20]. Some sample items are “In general, how would you say your health is?” for the physical well-being dimension; “Have you felt satisfied with your life?” for moods and emotions; and “Have you been happy with the way you are?” for self-perception. Cronbach’s alphas are ranging from 0.77 to 0.89 for the dimensions of the 52-item version. Except for the mood and bullying dimensions, higher values of the variables express a better health-related quality of life. KIDSCREEN questionnaires are psychometrically tested using data from a multicenter European study that included a sample of 22,827 children recruited in 13 countries [21].

Eating habits were evaluated using the Mediterranean Diet Quality Index for Children and Adolescents (KIDMED) [22, 23]. The KIDMED index was based on principles sustaining Mediterranean dietary patterns as well as those that undermine them (for example, “Every day I eat fruit or freshly squeezed fruit juice,” “Regularly once a day would consume fresh and cooked vegetables,” “I eat pasta and rice almost every day (5 or more per week”). The KIDMED score is calculated from 16 yes/no questions. Most of them concern the consumption of different food groups. For each “yes” response, one point is given to answers representing positive food habits (items 1–5, 7–11, 13, 15), and one point is subtracted for those representing negative food habits (items 6, 12, 14, 16). Three categories of adherence (good, average, and poor) were defined according to a score ≥ 8, between 4 and 7, and ≤ 3 [22, 23].

The validity of the KIDMED index is demonstrated by the evidence that a higher score is associated with the expected patterns of food and nutrient intake representative of a good quality diet (Cronbach’s alpha = 0.79, 95% CI 0.71–0.77).

## Statistical analysis

Statistical data analysis was performed using the SPSS software package (version 29, Armonk, NY: IBM Corp). Data are presented as mean ± SD or mean with 95% confidence interval (CI), and categorical variables are presented as counts and percentages. The Saphiro-Wilk test was used to assess the normality of the data distribution for continuous variables before parametric analyses. The *χ*^2^ test was used to assess associations between categorical variables. Global *p*-values were calculated by ANOVA followed by Tukey’s post hoc analysis. Spearman’s correlation coefficient was then used to assess associations between HRQoL, lifestyle habits with categories of PA, while differences between physical activity level categories were assessed by ANOVA followed by Tukey’s post hoc analyses. To account for the non-normal distribution of the variables, we performed sensitivity analyses using non-parametric statistical methods, which produced results consistent with the parametric tests (data not shown). Statistical significance was set at *p* < 0.05 for all tests.

## Results

### Socio-demographic characteristics of the study population

The total population consisted of 2819 healthy participants including 951 males (34%) and 1868 females (66%), distributed according to PA levels as follows: LPA 42%, MPA 57%, and HPA 1%. The population’s average age is 14.9 ± 3.7 years, of Italian nationality, from schools in Tuscany (48%), and Friuli Venezia Giulia (52%). Body mass index (BMI) in total population was 20.34 ± 5.94. Considering the PA categories, HPA subjects reported lower BMI as compared to LPA group (16.75 ± 9.98 vs 20.24 ± 5.75, *p* < 0.05) and MPA (16.75 ± 9.98 vs 20.46 ± 6, *p* < 0.05). No significant difference was found between LPA and MPA (20.24 ± 5.75 vs 20.46 ± 6). Most of the population engages in moderate physical activity (add 57%).

### Physical activity levels on HRQoL

Descriptive data of HRQoL in the total population stratified by PA levels are shown in Table 2. Belonging to the three PA categories, low, moderate, and high, affects all dimensions of the HRQoL.

More specifically, it was seen that in general practicing moderate or high PA offered a better perception of well-being: those who practiced moderate PA perceived better mood (*p* < 0.001), self-perception (*p* < 0.001), family relationship (*p* < 0.001), economic resources (*p* < 0.05), and social acceptance (*p* < 0.001); those who practiced high PA perceived better physical health (*p* < 0.001), psychological health (*p* < 0.001), autonomy (*p* < 0.001), and school learning (*p* < 0.001). The relationship with peers is perceived to be better by both MPA and HPA (*p* < 0.001).

Comparing MPA and LPA, the MPA group perceived better physical health (*p* < 0.001), psychological health (*p* < 0. 001), mood (*p* < 0.05), self-perception (*p* < 0.001), autonomy (*p* < 0.05), family relationships (*p* < 0.001), financial resources (*p* < 0.05), relationship with peers (*p* < 0.001), school learning (*p* < 0.001), and social acceptance (*p* < 0.05) (Table 1).

**Table 1:** HRQoL variables according to physical activity levels in total population

Variables	LPA (*n* = 1195)	MPA (*n* = 1605)	HPA (*n* = 19)	*p-value*	LPA *vs* MPA	LPA *vs* HPA	MPA *vs* HPA
Physical well-being	36.6 ± 6.3	45.6 ± 7.7	50.4 ± 11.0	< 0.001	< 0.001	< 0.001	0.01
Psychological well-being	33.6 ± 6.7	36.0 ± 6.4	41.5 ± 10.2	< 0.001	< 0.001	< 0.001	< 0.001
Mood/emotion	48.4 ± 10.5	49.2 ± 10.8	32.5 ± 17.8	< 0.001	0.05	< 0.001	< 0.001
Self-perception	40.0 ± 8.3	43.0 ± 8.8	42.0 ± 10.3	< 0.001	< 0.001	0.31	0.63
Autonomy	38.6 ± 8.8	40.8 ± 9.2	45.4 ± 15.0	< 0.001	0.003	0.001	0.03
Parent relationship	41.5 ± 10.4	43.9 ± 10.6	43.3 ± 16.8	< 0.001	< 0.001	0.47	0.81
Financial resources	48.2 ± 9.5	49.3 ± 9.4	47.5 ± 13.9	0.012	0.003	0.76	0.42
Peers	44.5 ± 9.9	46.8 ± 9.7	46.8 ± 17.7	< 0.001	< 0.001	0.33	0.99
School environment	41.0 ± 7.0	42.4 ± 7.2	46.3 ± 16.6	< 0.001	< 0.001	0.001	0.02
Social acceptance (Bullysm)	48.4 ± 10.5	49.2 ± 10.8	32.5 ± 17.8	< 0.001	0.05	< 0.001	0.001

Data given as mean ± SD (95% CI). Data on the KIDSCREEN-52 dimension are calculated as the mean *T*-scores according to KIDSCREEN Group. Global *p*-values were calculated by ANOVA test followed by Tukey’s post hoc analysis

*LPA* low physical activity, *MPA* medium physical activity, *HPA* high physical activity

Comparing the LPA group versus HPA, LPA is characterized by higher perceptions of mood (*p* < 0.001) and social acceptance (*p* < 0.001); conversely, HPA improves perceptions of physical health (*p* < 0.001), psychological health (*p* < 0.001), autonomy (*p* < 0.001), and school learning (*p* < 0.001) (Table 1).

Comparing MPA with HPA, MPA increased mood (*p* < 0.001) and social acceptance (*p* < 0.001), while HPA resulted in improved physical health (*p* < 0.05), psychological health (*p* < 0.001), and school learning (*p* < 0.05) (Table 1).

### Physical activity levels on lifestyle habits

Descriptive data of lifestyle habits in the study population stratified in low, moderate, and high PA are shown in Table 2. All the different behaviors are influenced by the different PA categories. In particular, MPA group showed a higher adherence to the Mediterranean diet (*p* < 0.001), a normal use of social networks (*p* < 0.001), and no eating disorders (*p* < 0.001), while physical activity, intended as time spent, was higher in the HPA group.

**Table 2:** Physical activity levels in lifestyle habits

Variables	LPA (*n* = 1195)	MPA (*n* = 1605)	HPA (*n* = 19)	*p-* *value*	LPA vs MPA	LPA vs HPA	MPA vs HPA
KIDMED	4.5 ± 2.6	5.7 ± 2.8	4.2 ± 3.8	< 0.001	< 0.001	0.6	0.03
PAQ-A	1.5 ± 0.3	2.7 ± 0.5	4.2 ± 0.3	< 0.001	< 0.001	< 0.001	< 0.001

Data given as mean±SD (95% CI). Global p-values were calculated by ANOVA test followed by Tukey’s post hoc analysis

*KIDMED* Mediterranean Diet Quality Index for children and adolescents, *PAQ-A* physical activity questionnaire for older children, *LPA* low physical activity, *MPA* medium physical activity, *HPA* high physical activity

Comparing LPA with MPA, LPA showed a reduction in extracurricular time (*p* < 0.001) and lower adherence to the Mediterranean diet (*p* < 0.001), whereas MPA in the area of social dependence reported normal use (*p* < 0.001), but a greater tendency to an eating disorder (*p* < 0.05). In contrast, comparing MPA with HPA only reduces time spent doing sports (*p* < 0.001) and MPA has a higher adherence to the Mediterranean diet.

### Spearman’s correlation analysis between HRQoL dimensions and the different levels of physical activity

There was a positive correlation between categories of PA levels and all HRQoL dimensions (Table 3). In particular, HPA was positively correlated with perceptions of physical (*p* < 0.001) and psychological health (*p* < 0.001), self-perception (*p* < 0.001), autonomy (*p* < 0.001), family relationships (*p* < 0.001), family economic status (*p* < 0.05), relationship with peers (*p* < 0.001), and school performance (*p* < 0.001). HPA was also positively correlated with adherence to the Mediterranean diet (*p* < 0.001) and time spent doing physical activity (*p* < 0.001) (Table 3).

**Table 3:** Spearman’s correlation coefficients and corresponding *p* values between HRQoL dimensions, diet, and physical activity levels

Variables	***R***	***p-value***
Physical well-being	0.58	< 0.001
Psychological well-being	0.20	< 0.001
Mood/emotion	0.03	0.10
Self-perception	0.17	< 0.001
Autonomy	0.13	< 0.001
Parent relationship	0.11	< 0.001
Financial resources	0.05	0.008
Peers	0.11	< 0.001
School environment	0.11	< 0.001
Social acceptance (Bullysm)	0.03	0.10
KIDMED	0.21	0.000

*KIDMED* Mediterranean Diet Quality Index for children and adolescents

## Discussion

The present study examined the possible effects of different PA intensities based on their frequency (low, moderate, or high) on health-related quality of life and lifestyle habits in a sample of high school Italian students.

Although numerous reports from the WHO, CDC, and other institution highlight the positive effects of PA in adolescents, both in preventive terms and by emphasizing its educational and social role, this study was focused on the relationship between PA intensity and health-related quality of life [24–28]. Our results show, in line with the latest ISTAT data, moderate PA, especially among boys, while girls tend to have low PA https://www.sport.governo.it/it/comunicazione-ed-eventi/studi-ricerche-ed-analisi/sport-attivita-fisica-sedentarieta/Looking in more detail at the relationship between PA categories and HRQoL, we have shown that moderate PA is associated with greater self-perception, higher mood, and richer social relationships, while high PA is associated with greater perception of health and well-being, both physical and mental, and better school learning. Furthermore, comparisons between the different categories show that mood is higher in conditions of low and moderate PA. It would therefore appear, based on our results, that different dimensions of quality of life are associated with PA in different ways. From the analysis of our results, it would appear that dimensions involving greater physical involvement are more associated with intense PA, whereas dimensions more related to psychological sphere seem to be associated with moderate PA and in the case of mood also with low activity. In terms of lifestyle habits, moderate PA is also associated with better nutrition.

In the literature, the effect of PA on health-related quality of life in adolescents had already been studied [29], but rarely to assess whether there were differences based on the amount of PA performed. One study in particular by Merglen and colleagues went to assess this by showing that maximum well-being scores were reached around 14 h/week of sports practice and that the association was reversed when exceeding 17.5 h/week [11]. In another study, the participation to three-to-five different sport setting was associated with higher well-being [11].

More generally, our results demonstrated correlations between HRQoL dimensions, lifestyle habits, and performing PA. The more PA was performed, the more adolescents reported an increase in perceptions of physical and psychological health, self-perception, autonomy, family relationships, socioeconomic status, peer relationships, and academic achievement [30].

The results of this study reveal the importance of sports and their protective role, not only for the prevention of chronic diseases, but in the perception of well-being in adolescence and the adoption of healthy behaviors. More generally, PA is seen in adults as a preventive therapy in the healthy population and as an actual therapy when cardiovascular diseases are present. PA, when also combined with proper nutrition and good sleep quality, has important benefits on the mental health and well-being of young people [31]; in this sense, PA becomes a key factor for mental and physical balance in adolescence. One possible explanation for such a sharp division between the physical and psychological components associated with PA could depend on the fact that moderate PA at this age is important for socializing, spending free time, and certainly taking care of healthy activities, but perhaps indirectly. The focus is more associated with the strengthening of these dimensions, and the results confirm this. The question is different for the intense PA, which is carried out either at a competitive level or for special physical and performance reasons. A specific role is thus deduced in the physical variables. However, the competitive role, if not properly managed, could reduce the social and educational effects of sport, and go further to create fragility, low self-esteem, and in the long run, dysfunctional behavior. This was supported by the fact that the mood in boys who engaged in strenuous physical activity was lower and so was social acceptance.

In the literature, the evidence on the role of physical activity in promoting well-being is clear, improving internalizing and externalizing symptoms and reducing depression, anxiety, and substance use disorders in youth [32, 33]. More controversial is the finding on the effects of the intensity of sporting activity on health and well-being. Same results suggest that higher levels of PA lead to greater mental well-being in youths, while a recent study has demonstrated that low and moderate programs significantly reduced depression and anxiety, whereas high PA did not induce any effects [34, 35]. Another important aspect that emerged from the data is the link between high PA and learning. On this point, too, the literature is not very clear. On the contrary, a positive relationship between aerobic fitness and academic achievement is well documented in school-age children [36]. One possible explanation of this association is related to the influences of aerobic activity on hippocampal volume, a cerebral region involved in mnemonic processes [37].

To date, sports play a decisive role in our culture and have a strong educational value. Accordingly, the importance of sports has also been recognized within the Italian Constitution, where in 2023, the following paragraph was added to Article 33: “*The Republic recognizes the educational, social and promotion of mental and physical well-being value of sporting activity in all its forms*” (https://www.senato.it/istituzione/la-costituzione). Thus, in addition to having a preventive role for the development of diseases, PA assumes a more complex role in the individual’s concept of health, traditionally defined by the WHO as “*a state of complete physical, mental and social well-being and not simply the absence of disease*” [10]. It allows young people to become accustomed to structuring their time, controlling their emotional reactions, and respecting their commitments and time requirements. Sports have both an educational and social function. While it allows adolescents to focus on their mental and physical health and the goals to be achieved, it also functions as a social aggregator.

However, the intense activity seems to enhance, according to our results, the cognitive component, here expressed in terms of school performance. To our knowledge, there are no studies in the literature that have demonstrated the results agree with ours; in fact, there even seems to be data to the contrary [3, 38].

The study has some limitations. The first is related to the fact that the data collected came from two specific, geographically distant areas. Therefore, these data may not be generalizable to all adolescents who participate in sport or to all types of sport. The questionnaire used, the PAQ-A, seemed to be the most suitable questionnaire for the type of sample considered within this study, although not consider sedentary people in the reference population, but to our knowledge, there are no other commonly used and standardized tests that include those who do not play sports in the reference population. The last limitation might concern the use of questionnaires (self-perceptions) and the risk of “fatigue” in the compilation, given the length of the instruments and the age of the population.

## Conclusion

The results of this study suggest that there is an association between the frequency of PA and some dimensions of HRQoL and that this association is more pronounced in adolescents who engage in more PA during the week. Our data demonstrated the importance and need to implement strategies to promote sports practice in the adolescent population that also take into account psychological dimensions. These factors should be considered when designing programs to promote health and well-being in adolescents.

## Data Availability

No datasets were generated or analysed during the current study.
